# Memory reactivation during rest forms shortcuts in a cognitive map

**DOI:** 10.1038/s41598-025-06742-y

**Published:** 2025-07-09

**Authors:** Cal M. Shearer, Annalise B. Rawson, Helen C. Barron, Jill X. O’Reilly

**Affiliations:** 1 Department of Experimental Psychology, University of Oxford, Oxford, UK; 2 Medical Research Council Brain Network Dynamics Unit, Nuffield Department for Clinical Neurosciences, University of Oxford, Oxford, UK; 3 The Oxford Centre for Integrative Neuroimaging, FMRIB, John Radcliffe Hospital, University of Oxford, Oxford, UK

**Keywords:** Neuroscience, Cognitive neuroscience, Learning and memory

## Abstract

Efficient and flexible cognition relies upon cognitive maps—representations of concepts and the relations between them. Cognitive maps integrate relations that were learned separately into a cohesive whole. Memory reactivation during rest and sleep may contribute to cognitive map formation in two ways: by simply strengthening memories for directly experienced relations, or by reorganising concepts and creating new relations that capture the underlying structure. We designed a multi-stage learning task to test whether reactivation during rest is involved in restructuring memories as opposed to simply consolidating what was experienced. We causally manipulated memory reactivation during rest using awake, contextual targeted memory reactivation. We found that promoting memory reactivation during rest qualitatively reorganises the cognitive map by *forming ‘shortcuts’ between* events which have not been experienced together. These shortcuts in memory extend beyond direct experience to facilitate our ability to make novel inferences. Using a series of control tests we show that inference performance cannot be explained by quantitative strengthening of the experienced component links. Interestingly, we show that representing a shortcut may come with limitations, as shortcuts cannot be readily updated in response to rapid changes in the environment. Together, these findings reveal how memories are reorganised during awake rest to construct a cognitive map of our environment, while highlighting the constraints set by a trade-off between efficient and flexible behaviour.

**Supplementary Information:**

The online version contains supplementary material available at 10.1038/s41598-025-06742-y.

## Introduction

It is proposed that we understand our environment by constructing a cognitive map^1^. Within a cognitive map we can represent events in the external world and the relations between them. Importantly, the cognitive map is thought to go beyond the sum of its parts, by ‘knitting together’ events and relations that have been experienced separately and by building entirely new relations that have not been directly experienced. This allows a cognitive map to not simply aid navigation along familiar paths, but can also support navigation into unknown territories, whether spatial or more abstract.

As a simple example, if an observer has seen the pairwise associations A → B and B → C, they can infer the *unobserved* association A → C^2^. There are two ways in which the inferred A → C relationship could be represented: as a chain of learned associations (A → B → C), or by formation of a new ‘shortcut’ or direct relation (A → C). The formation of a new shortcut (A → C) would represent a *qualitative* restructuring of memories, to build an entirely new relation that has not been directly experienced. The ability to make such qualitative changes in the organisation of knowledge is considered an essential feature for a cognitive map that supports flexible behaviour^3–6^. Here we investigate the hypothesis that offline periods contribute to the qualitative restructuring of memories. Specifically, we predict that while associative memories are formed during active learning, memory reactivation of these associations during periods of rest and sleep may introduce ‘shortcuts’ into the cognitive map, to support flexible behaviour.

The process of constructing a cognitive map is thought to be facilitated by offline periods of rest/sleep^7–14^. During these periods, memories for previous experiences are reactivated or ‘replayed’^15–17^. Replay involves temporally structured spiking activity that recapitulates previous waking experience^18–20^ to facilitate memory consolidation^15–17,21^ and subsequent decision-making^22^. Importantly, growing evidence suggests replay may play a particular role in extending the cognitive map beyond previous waking experience to anticipate upcoming events^23,24^, restructure knowledge^25^, or even “join-the-dots” between spatial trajectories or events that were not directly experienced together^26–28^. Here, we explicitly test the hypothesis that offline periods of rest *qualitatively* reorganise memories, rather than *quantitatively* changing the strength of associations that were actually experienced. We define qualitative re-organisation as the creation of new inferred links (or shortcuts) that are independent of experienced associations, thus forming a cognitive map that is more than the sum of its parts.

To test our hypothesis, we designed a multi-stage inference task adapted from a protocol previously implemented in humans and mice^26^. We causally manipulated memory reactivation during awake rest using contextual auditory TMR^29,30^. Consistent with our predictions, we demonstrate that biasing memory reactivation during awake rest improves the ability to infer novel relationships between sensory cues. Furthermore, using behavioural and physiological (eye-tracking) data, we show that this improvement in inference is driven by the formation of novel shortcuts between indirectly linked cues. As a result of these shortcuts, participants are less reliant on memory of intermediary associations (A → B and B → C) since they can instead use the direct, yet unobserved, shortcut formed during awake rest (A → C) to inform their decisions. However, although beneficial for inference, we show that building cognitive shortcuts may come with limitations: when the component associations are modified (e.g. B → C changes to B → C*), this change is not immediately transferred to the shortcut, leaving the old shortcut (A → C) to compete with the updated chain of associations (A → B, B → C*).

Together our findings show that memory reactivation enhances inferential choice through the formation of shortcuts between indirect associations. However, these shortcuts cannot be rapidly updated. We thus demonstrate that memory reactivation during awake rest contributes to constructing a cognitive map that is more than the sum of its parts, allowing for efficient behaviour that is limited in flexibility.

## Results

### Task design and learning performance

**Fig. 1:**
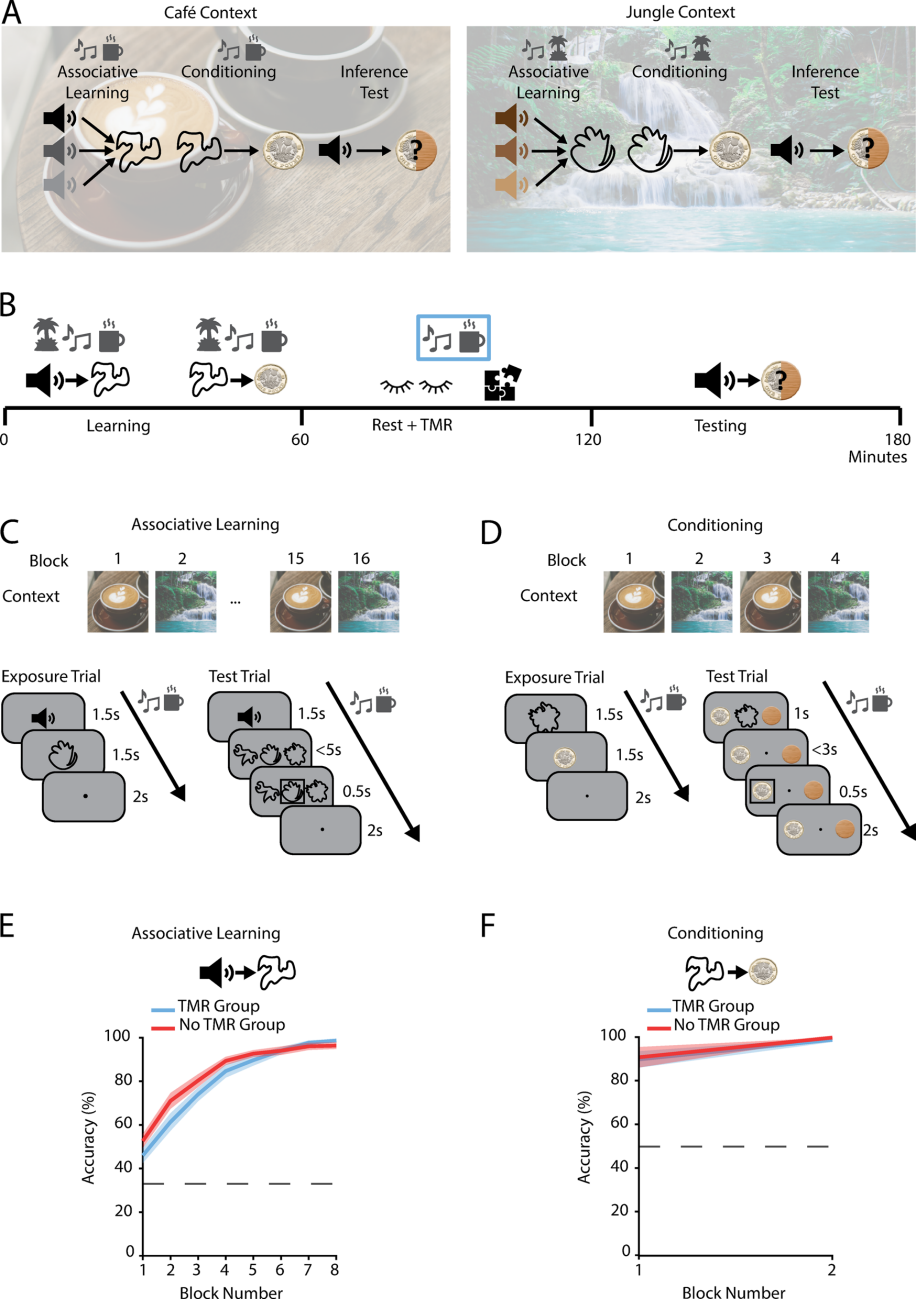
Inference task design and learning performance. (**A**) Three-stage inference task adapted from a previous protocol^22^. In learning phase 1, participants learnt to associate auditory cues with visual cues (‘Associative Learning’), with a many-to-one mapping. In learning phase 2, participants then learnt to associate visual cues with an outcome (rewarded—pound coin; neutral—wood coin) (‘Conditioning’). During the ‘Inference Test’, the auditory cues were presented in isolation, and we assessed participants’ ability to infer the value of any given auditory cue. Cues were divided into two sets, each grouped according to the contextual background music that played throughout learning (set 1: café context—left; set 2: jungle context—right). (**B**) Timeline of the task design in minutes. The task was split into three phases—learning, awake rest, and testing. During each block of the learning phase, the contextual background music for the relevant context was played. During the awake rest period, the contextual background music for only one set of cues was played to bias memory reactivation towards cues in this set (TMR group). After the awake rest period, participants were tested on their ability to infer associations between the auditory cues and the outcome cues. (**C**, **D**) Structure of the associative learning phase (**C**) and conditioning phase (**D**). Top: learning trials were blocked by context and alternated between the two contexts using the contextual background music. Bottom: Example trials (schematic). Blocks of trials consisted of both exposure trials (left) and test trials (right). (**E**, **F**) Behavioural performance during the associative learning (**E**) and conditioning (**F**) phases (mean accuracy ± SEM). Participants only proceeded to the inference test if they reached criterion (> 85% performance accuracy for each group in both associative learning and conditioning). There was no significant difference in the maximum accuracy reached between sensory cues in the two contexts (café vs jungle; *p* = 0.385, paired bootstrapped test, two-tailed) or between the group of cues that were subsequently subject to TMR and no TMR (TMR group vs no-TMR group; *p* = 0.185, paired bootstrapped test, two-tailed). Background images by Rifqi Ramadhan on Pexels.com (jungle) and Photo by shche_ team on Unsplash (coffee), with permission.

To investigate the formation of shortcuts in a cognitive map, we designed an inference task (Fig. 1A,B). The inference task was adapted from a previous inference protocol implemented in humans and mice^26^ that leveraged a sensory preconditioning paradigm^2^. The inference task included three stages.

#### Learning phase

In the first learning stage (A → B), participants learnt to associate pairs of auditory and visual cues (‘associative learning’), with a many-to-one mapping from auditory to visual cues. The presentation of task cues was blocked into two groups, distinguished by different contextual background music that played throughout learning (either café or jungle music). One of these contextual background tracks was later played during rest to bias memory reactivation to this group of cues (TMR manipulation). After training, participants’ mean accuracy on the associative learning trials was 96.67% (SD = 7.41%), with no significant difference in the maximum accuracy reached between sensory cues in the two contexts (café vs jungle: *p* = 0.385, paired bootstrapped test, two-tailed) or between the group of cues that were subsequently subject to TMR and the group of cues that were not (TMR group vs no-TMR group: *p* = 0.185, paired bootstrapped test, two-tailed); Fig. 1E).

In the second learning stage (B → C) of the inference task, participants learnt to associate the visual cues with either a rewarding outcome (represented by a pound coin) or a neutral outcome (represented by a wooden coin) (‘conditioning’). As in the associative learning phase, contextual background music was played throughout learning. After training, participants achieved a mean accuracy of 99.50% on the conditioning trials (SD = 2.50%) (Fig. 1F).

#### Rest and TMR

Following the second learning phase, we applied a TMR manipulation^31–34^ to causally manipulate memory reactivation by biasing each participants resting memory reactivation towards a randomly allocated subset of cues. Participants underwent the TMR manipulation during a period of awake rest^29^ (60 min), after learning stage 2 (i.e., after completing the associative learning and conditioning phases of the task), but before performing the inference test. Throughout this awake rest period, we played the contextual background track associated with cues in one of the two groups (i.e., either café music or jungle music, fully counterbalanced across participants) whilst participants completed a relaxing task unrelated to the learning task (namely, they completed a jigsaw puzzle). We predicted that this manipulation should bias the content of memory reactivation towards one group of cues (A, B, C) and any learned associations within that group (i.e. A-B, B-C). Moreover, we hypothesised that if memory reactivation driven by TMR also facilitates the construction of a cognitive map that is more than the sum of its parts, memory reactivation should build shortcuts between cues that have not been directly experienced together (e.g. A-C), to facilitate subsequent inference across cues in the TMR group.

#### Test phase

Importantly, auditory cues were never paired with outcome cues, providing an opportunity to assess evidence for an inferred relationship between these indirectly related cues. Accordingly, participants performed an ‘inference test’. In the inference test, we presented auditory cues in isolation, without visual cues or outcome cues, and without the contextual background music. In response to these isolated auditory cues, we measured evidence for inference by asking participants which outcome was associated with each auditory cue (i.e., we tested the A → C relationship), despite the fact that they had never directly experienced this pairing.

During the test phase, the inference task was performed twice, before and after a ‘value-flip’ manipulation. During the value-flip (B → C*), a subset of the associations between visual cues and outcomes were ‘Flipped’, so that previously rewarded visual cues became neutral and vice versa. Participants learned these new associations, with the contextual background soundtrack playing, as in learning phase 2. However, this value-flip was not followed by a period of rest. Instead, participants immediately repeated the inference task.

The purpose of the value-flip manipulation was to investigate to what extent learning of the updated associations B → C* was transferred to the inferred shortcut A → C. We reasoned that if participants relied upon a chain of directly observed associations A → B → C to support inference, learning of the new link B → C* would result in a new chain A → B → C*. However, if participants use an inferred shortcut A → C, formed during awake rest, this shortcut would not be immediately updated in response to learning the new B → C* associations. Instead, A → C might only be updated to A → C* during another period of awake rest.

### TMR improves inference performance

**Fig. 2:**
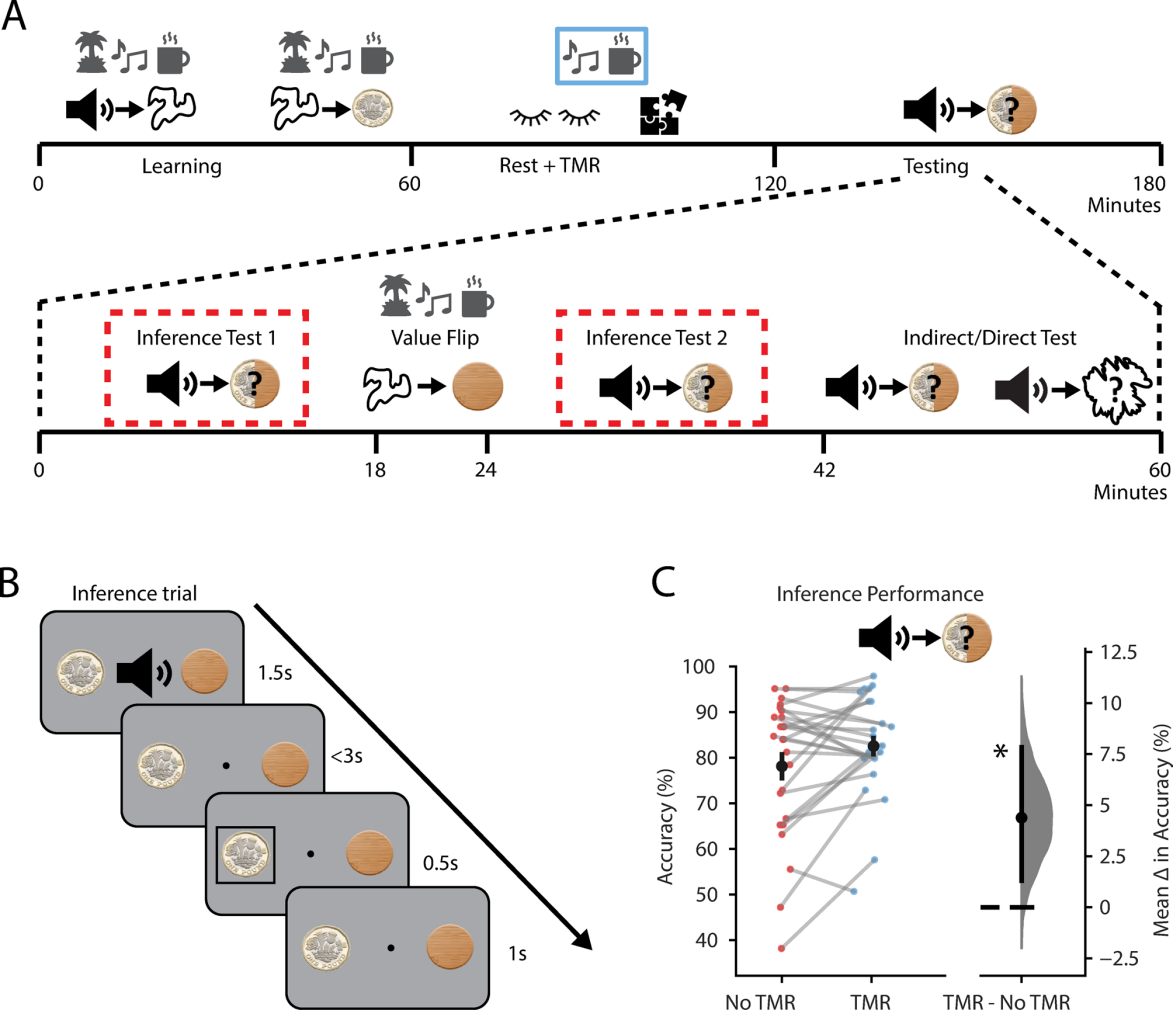
TMR improves inferential choice. (**A**) Structure and timings of the testing phase of the task. Tests highlighted in red were combined to examine the effect of TMR on the ability to infer an association between the auditory cues and the outcome. The remaining stages (‘Value Flip’ and ‘Indirect/Direct Test’) are detailed in Figs. 3 and 4. (**B**) Schematic: Example trial in the inference tests (I.e., ‘Inference Test 1’ and ‘Inference Test 2’). (**C**) Inference test performance. Participants were significantly better at inferring the correct outcome for auditory cues in the TMR group (*p* = 0.013, paired bootstrapped test, one-tailed). No significant effect was observed in reaction time data, where reaction times were not speeded. Left: raw data points for accuracy on auditory cues in the No TMR group (red; left) and TMR group (blue; right); each data point: mean accuracy for a given participant; black dot, mean; black ticks ± SEM. Right: difference in mean inference accuracy between auditory cues in the No TMR and TMR groups shown using bootstrap-coupled estimation (DABEST) plots^48^. Effect size for the difference between No TMR and TMR groups was computed from 100,000 bias-corrected bootstrapped resamples^52^: black dot, mean; black ticks, 90% confidence interval; filled-curve, sampling-error distribution. *indicates *p* < 0.05.

After the period of awake rest, participants performed the inference test (Fig. 2A,B). Here, we presented the auditory cues in isolation and tested participants’ ability to infer a relationship with the corresponding outcome cue (rewarding or neutral). To assess the effect of TMR on inference, we compared participants’ accuracy in response to auditory cues in the TMR and no TMR groups.

Participants were better at inferring the correct outcome for auditory cues in the TMR group than the non-TMR group (*p* = 0.013, Fig. 2C—this and all subsequent p values, unless otherwise noted, from paired bootstrap tests, one-tailed, as described in *methods*). The inference task was carried out both in isolation, and in the presence of two distractor tasks (a one-back task and a semantic judgement task, see Methods). The distractor tasks were designed to increase the load on visual working memory or general cognitive resources respectively, to modulate task difficulty and avoid ceiling effects when inference was performed in isolation. Specifically, we predicted that including a visual working memory distractor should impair inference if participants rely on recall for the intermediate visual cue (Figure S1A). However, participants were better at inferring the correct outcome for auditory cues in the TMR group under all three variants of the inference task (*p* = 0.013, no distractor task; *p* = 0.031, visual working memory task; *p* = 0.031, non-memory task; Fig. S1), with no significant difference in the TMR effect between conditions (*p* = 0.237, no distractor task vs memory task; *p* = 0.741, no distractor task vs non-memory task; Fig. S1). Hence, hereafter results are pooled across the three distractor conditions. Overall, participants were better at inferring the correct outcome for auditory cues in the TMR group (*p* = 0.013, Fig. 2C). This result demonstrates that biasing memory reactivation during periods of rest with awake, contextual TMR facilitates inferential choice.

### TMR creates a shortcut in memory between indirectly linked cues

Having established that TMR facilitates inference of the unobserved A → C link, we went on to probe the nature of that facilitation. Specifically, we asked: do participants build a shortcut (A → C) that is separate from the original chain of learned associations (A → B, B → C), or do they instead strengthen the component associations in the original chain (A → B, B → C)? We reasoned that if TMR facilitates inference by building a new shortcut between the auditory and outcome cues (A → C), the resulting cognitive map may be less amenable to flexible updating when associations between cues change.

Specifically, if the visual-outcome association B → C changes to B → C*, the correct chain of associations to support inference is now A → B and B → C*. If participants perform inference using the chain of learned associations (A → B, B → C) then the inference A-B-C* should immediately reflect any update in the component to B → C*. By contrast, if a direct shortcut (A → C) was constructed during awake rest, this would not be automatically updated to A → C* and behaviour may not account for the changes from B → C to B → C*.

### ‘Flipping’ the component associations negates the behavioural benefit of TMR

**Fig. 3:**
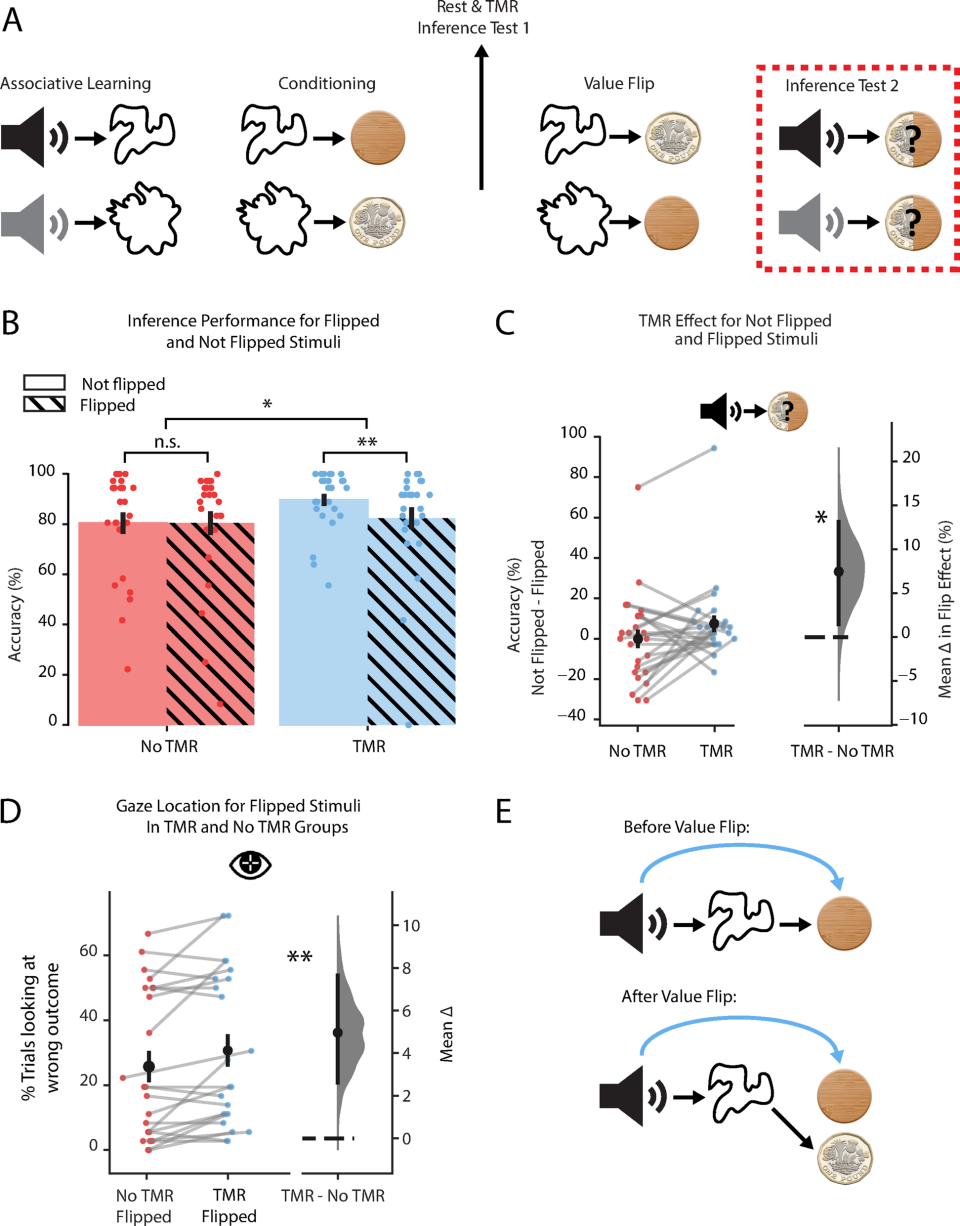
TMR creates a shortcut which cannot be readily updated. (**A**) Schematic illustrating the value flipping manipulation. After the learning and awake rest phases, the outcome associated with half of the visual cues was flipped (‘Value Flip’). During Inference Test 2 (highlighted in red), we tested to see if participants could correctly infer the new outcome that was indirectly associated with the auditory cue. (**B**) Inference test performance after the value flip manipulation. A significant interaction was observed in inference accuracy in response to auditory cues in the Flipped vs. Not Flipped condition for the TMR vs. no TMR group (*p* = 0.025, paired bootstrap test, one-tailed). The predicted directionality of this effect was confirmed using post-hoc tests. Within the TMR condition, a significant difference in inference accuracy was observed between the Flipped and Not Flipped cues (*p* = 0.003, post-hoc paired bootstrap test, one-tailed), while no significant difference was observed within the no TMR condition (*p* = 0.490, post-hoc paired bootstrap test, one-tailed). Within the Not Flipped condition, but not in the Flipped condition, a significant difference in inference accuracy was observed for the TMR vs. no TMR cues (Not Flipped: *p* = 0.001; Flipped: *p* = 0.234; post-hoc paired bootstrap test, one-tailed). No significant effects were observed in reaction time data (reaction times were not speeded). Bars: mean percentage accuracy for No TMR (red; left two bars) and TMR (blue; right two bars) groups split by whether the visual cue-outcome association was Not Flipped (plain; left bar of each group) or Flipped (hatched; right bar of each group); black ticks: ± SEM; each data point: mean accuracy for one participant. (**C**) As shown in B, a significant interaction was observed in inference accuracy in response to auditory cues in the Flipped vs. Not Flipped condition for the TMR vs. no TMR group (*p* = 0.025, paired bootstrap test, one-tailed). No significant effect was observed in reaction time data (reaction times were not speeded). Left: raw data points for No TMR cues (left) and TMR cues (right); each data point: mean effect of flipping (Not Flipped–Flipped accuracy) for a given participant; black dots, mean; black ticks ± SEM. Right: difference in means between TMR and No TMR cues shown using bootstrap-coupled estimation (DABEST) plots as in Fig. 2C: black dot, mean; black ticks, 90% confidence interval; filled-curve, sampling-error distribution. (**D**) For auditory cues where the visual cue-outcome association was flipped, gaze data revealed a significant difference in percentage of trials spent looking at the wrong outcome for auditory cues in the TMR compared to the No TMR group (*p* = 0.002, paired bootstrap test, one-tailed). Analysis applied to correct trials only. Left: raw data points for No TMR group (red; left) and TMR group (blue; right); each data point: mean percentage of trials looking at wrong outcome for a given participant; black dots, mean; black ticks ± SEM. Right: difference in means between No TMR and TMR groups shown using bootstrap-coupled estimation (DABEST) plots as in Fig. 2C: black dot, mean; black ticks, 90% confidence interval; filled-curve, sampling-error distribution. See Figure S3 for equivalent plot for Not Flipped cues. (**E**) Schematic showing how a shortcut may conflict with a chain of direct links after the value-flip manipulation. Top: before value-flip, both the chain of direct links (black arrows) and the shortcut formed by TMR (blue curved arrow) lead to the same outcome. Bottom: After value-flip, the chain of direct links has been updated but now conflicts with the shortcut which cannot be rapidly updated. *indicates *p* < 0.05; ** indicates *p* < 0.01; n.s. indicates no significant difference.

To test this hypothesis, after the first inference test, we manipulated the association B → C (visual-outcome), such that the outcome associated with a subset of visual cues was reversed (value-flip manipulation, B → C*). Participants were exposed to this value-flip manipulation across a relatively brief 6-min task (Fig. 2A), before being asked to immediately perform the inference test once more, to test their ability to flexibly update the inferred value of auditory cues (from A → C to A → C*) (Fig. 3A). Critically, if TMR creates a shortcut (A → C) between auditory and outcome cues that is separate to the chain of learned auditory-visual-outcome associations (A → B, B → C), then an update in the visual-outcome leg (B → C*) should not transfer to the auditory-outcome (A → C) shortcut. Therefore, to make correct inferences, changes to the visual-outcome (B → C*) mapping would require participants to draw on the complete chain of learned associations (A → B, B → C*) for cues in both the TMR and no TMR groups, negating the benefit of TMR. In addition, representation of an auditory-outcome (A → C) shortcut should compete with flexible updating in response to changes to the visual-outcome (B → C*) mapping. Taken together, we predict an interaction in the effect of TMR and the value-flip manipulation on inference accuracy.

We observed evidence for a significant interaction between the effect of TMR and the value flip manipulation on inference accuracy (*p* = 0.025, Fig. 3B,C). We note that this interaction analysis effectively controls for the difference in baseline inference accuracy between cues in the TMR and no TMR groups. Any detrimental effect of value flipping will operate upon this baseline accuracy. We also note that overall performance accuracy remained high, suggesting that even when a detrimental effect of value flipping was observed, on the majority of trials participants were still able to use the complete chain of learned associations to perform inference (A → B, B → C*).

Using a series of post-hoc paired tests, we further demonstrate that the direction of the interaction between TMR and the value-flip manipulation was as predicted. Namely, within the TMR condition, which inherently controls for the effect of TMR on inference accuracy, a significant effect of the value-flip manipulation can be observed (*p* = 0.003, Fig. 3B). Moreover, for the Not Flipped but not the Flipped cues, a significant effect of TMR was observed (Not Flipped: *p* = 0.001; Flipped: *p* = 0.234). Therefore, for visual-outcome mappings (B → C) where there was no change in the association (Not Flipped associations), participants were better at inferring the correct outcomes for auditory cues in the TMR compared to the no TMR group (*p* = 0.001, Fig. 3B,C). However, for flipped associations (B → C*), there was no significant difference between cues in the TMR and no-TMR groups (*p* = 0.234, Fig. 3B,C). Taken together, these results suggest that the value flip manipulation impairs inference, when controlling for the effect of TMR, and the benefit of TMR is negated by the value-flip manipulation. This is consistent with the proposal that, after rapid updating in the visual-outcome (B → C*) mapping, participants had to perform inferential choice by drawing on the complete chain of learned associations (A → B, B → C*), rather than the shortcut (A → C), thus negating the benefit of TMR.

### Gaze data suggest that the outdated shortcut competes with the flipped association

Next, we reasoned that if the outdated shortcut A → C continues to be represented, it might in fact compete for behavioural control with the modified chain of learned associations (A → B, B → C*). Such competition would strengthen the conclusion that the inferred shortcut A → C exists as a separate entity to the chain of observed associations (evidence that rest qualitatively changes the cognitive map). Therefore, during the inference test performed after the value-flip manipulation, we used eye-tracking data to ask whether forming a shortcut (A → C) biases participants to look at the previously associated (but now incorrect) outcome (C) in the inference test (Fig. 3E). To do this, we took all trials where participants performed correct inference and then calculated the percentage of these trials where participants looked at the incorrect outcome, noting that for Flipped cues the incorrect outcome corresponded to the outcome cue (C) that was previously correct prior to the value-flip manipulation.

We hypothesised that for auditory cues where the visual-outcome association was flipped (B → C*), in the TMR condition participants may still retain a shortcut (A → C) from the auditory cue to the (now incorrect) outcome (Fig. 3E). In these cases, participants would be more likely to look at outcome C rather than C* during the decision process. Thus, relative to auditory cues in the TMR group, we predicted that this effect should be lower, or absent for cues in the no-TMR group where the shortcut (A → C) was weaker or not formed. This was indeed the case. For Flipped cues, participants were significantly more likely to look at the incorrect outcome cue (C) in the TMR group compared to the no TMR (*p* = 0.002, Fig. 3D; see Figure S3 for Not Flipped cues). This suggests that when a shortcut (A → C) forms due to TMR, knowledge of this shortcut competes with knowledge of relevant updated associations. This competitive effect supports the view that the shortcut (A → C) is separate from the chain of learned associations (A → B, B → C). Consequently, TMR may be considered to support efficient behaviour by facilitating inference through the formation of a shortcut between auditory and outcome cues even when these cues have not been directly experienced together. However, building a shortcut in the underlying cognitive map appears to have limitations—the shortcut cannot be rapidly updated in response to environmental change.

### Gaze data show that participants use intermediary cues more for non-TMR associations

**Fig. 4:**
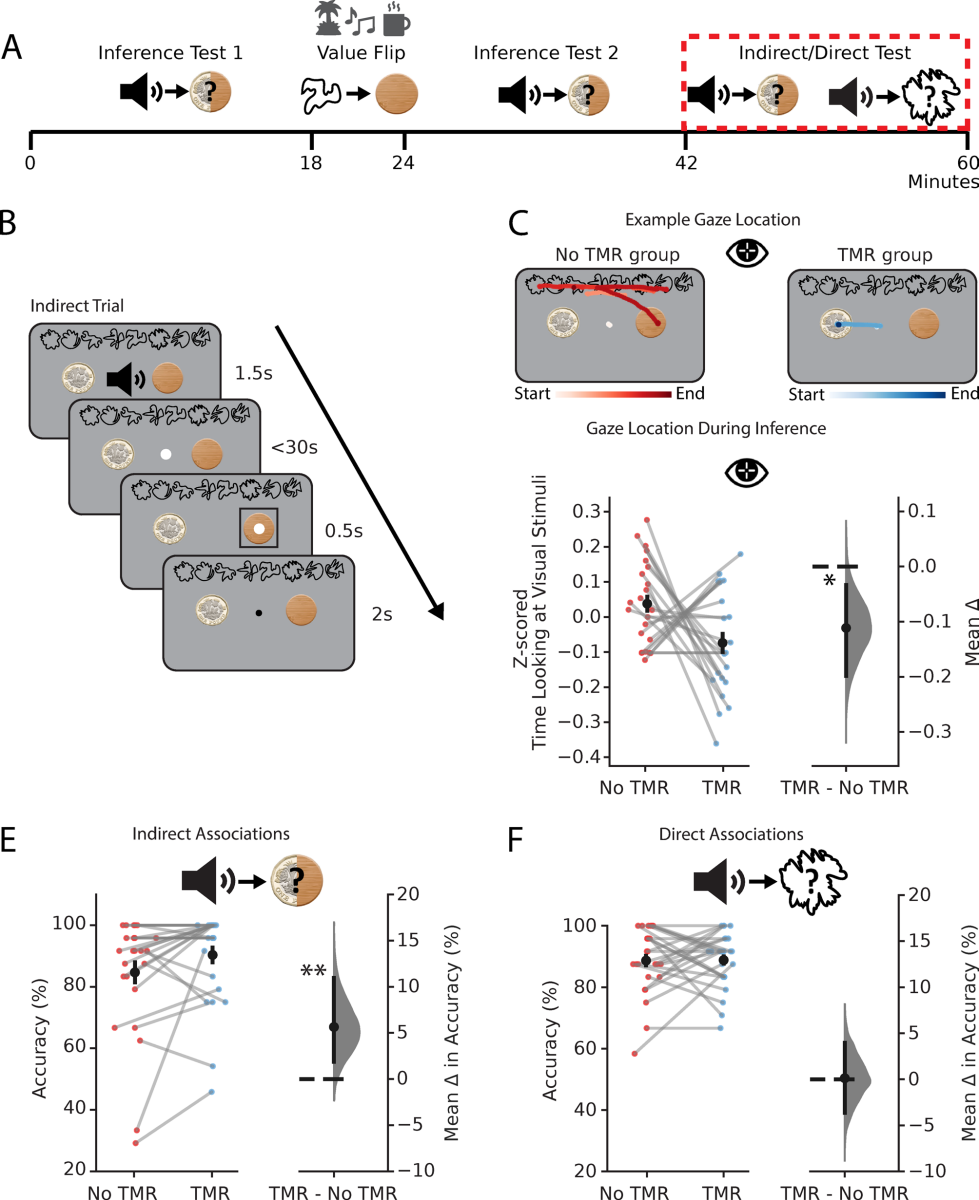
TMR Creates a shortcut which does not rely on Directly Learnt Associations (**A**) Structure of the testing phase of the task. Data presented in (**C**–**F**) is from the ‘Indirect/Direct Test’ highlighted in red. In the indirect/direct test, participants were tested on both the indirect (auditory–outcome) and direct (auditory–visual) associations for each auditory cue. (**B**) Schematic: example indirect trial (schematic) where auditory cues were now presented while the visual cues were on screen. Participants were required to infer the outcome cue in response to the auditory cue. Direct trials followed the same structure except, after hearing the auditory cue, participants had to choose the associated visual cue rather than the associated outcome. (**C**) Gaze location from eye tracking data acquired during indirect trials in the indirect/direct test. Participants spent significantly less time looking at the visual cues in response to auditory cues in the TMR group (*p* = 0.014, paired bootstrap test, one-tailed). Analysis applied to correct trials only. Top: example gaze trajectories from a single indirect trial for one participant in response to an auditory cue from the No TMR group (red; left) and an auditory cue from the TMR group (blue; right). Darker colours indicate later time in the trial. Bottom left: raw data points for No TMR group (red; left) and TMR group (blue; right); each data point: mean z-scored time spent looking at visual cues during inference (correct trials only); black dots, mean; black ticks ± SEM. Bottom right: difference in means between No TMR and TMR groups shown using bootstrap-coupled estimation (DABEST) plots as in Fig. 2C: black dots, mean; black ticks, 90% confidence interval; filled-curve, sampling-error distribution. (**D**, **E**) The effect of TMR on indirect associations (**D**) and direct associations (**E**). There was a significant effect of TMR on accuracy for indirect associations (*p* = 0.009, paired bootstrap test, one-tailed, for both Flipped and Not Flipped cues) but not for direct associations (*p* = 0.470 paired bootstrap test, one-tailed, for both Flipped and Not Flipped cues). No significant effect was observed in reaction time data, where reaction times were not speeded. Left of each panel: raw data points for No TMR group (red; left) and TMR group (blue; right); each data point: mean accuracy for a given participant; black dots, mean; black ticks ± SEM. Right of each panel: difference in means between No TMR and TMR groups shown using bootstrap-coupled estimation (DABEST) plots as in Fig. 2C: black dot, mean; black ticks, 90% confidence interval; filled-curve, sampling-error distribution. *indicates *p* < 0.05, **indicates *p* < 0.01.

In a final set of analyses, we again used eye-tracking data to ask whether TMR facilitates inference by creating a shortcut in memory between the auditory and outcome cues. We reasoned that if a shortcut (A → C) is represented, we would expect participants to be less likely to use the intermediary visual cues (B) to help make inferential choices for cues in the TMR group. To test this, we analysed eye-tracking data acquired during correct trials on a third inference test (‘indirect’) and during a memory test for learned associations (‘direct’) (‘indirect/direct’ test, Fig. 2A, 4A,B). During the first half of this testing phase, participants again had to decide whether the auditory cues were associated with the rewarding or neutral outcome (inference test, ‘indirect’). Replicating our previous findings, we found that participants were again significantly more accurate at inferring the correct relationships for auditory cues in the TMR group compared to the no TMR group (*p* = 0.009, Fig. 4D). However, unlike in the previous inference tests, all of the intermediary visual cues were now presented on the screen (Fig. 4B). We predicted that if participants had formed a shortcut between auditory and outcome cues, they would be less likely to look at the visual cues to help them infer a value for each auditory cue. Thus, despite the value-flip manipulation, we expected cues in the TMR condition (relative to the no TMR condition) to be linked via a short-cut (A → C) that influences gaze location, for both the Flipped and Not Flipped cues, by reducing time spent looking at the intermediary visual cue (B). Consistent with our prediction, on correct trials participants spent less time looking at the visual cues for auditory cues in the TMR group compared to the no TMR group (*p* = 0.014, Fig. 4C). Therefore, for cues in the TMR group, there was a reduced need to look at the intermediary visual cue to help make a correct inference. Together with our previous findings, this result suggests that TMR facilitates the formation of a memory shortcut between the indirectly linked auditory and outcome cues.

### The effect of TMR on inference is not explained by improved memory for directly learnt associations

Finally, we asked whether awake contextual TMR also facilitates memory accuracy for directly associated cues. Previous work has shown that TMR protocols that use punctate auditory cues improve memory for directly learnt associations^35^. However, since our TMR protocol uses contextual cues, we wanted to investigate whether this protocol would have similar or different effects on memory. During the indirect/direct test (Fig. 4A,B), participants were also given a memory test for learned associations between auditory and visual cues (‘direct associations’). By contrast to performance on the inferred relationships between auditory and outcome cues (‘indirect associations’) reported above (Fig. 4D), when participants were tested on direct associations, they showed no significant difference in accuracy for auditory-visual (A → B) cue mappings in the TMR group compared to the no TMR group (*p* = 0.470, Fig. 4E).

Since participants were trained to ceiling on the direct associations, it is possible that we did not see an effect of TMR due to ceiling performance. To investigate this potential confound, we performed a split based on combined performance for TMR and no TMR groups, and applied this to both the indirect association (inference) test (in which we saw an effect of TMR) and direct association test (in which we did not see an effect of TMR) (Figure S2). For direct associations, even for participants who were not at ceiling, we continued to observe no significant effect of TMR (*p* = 0.389, top 50%; *p* = 0.368, bottom 50%), suggesting that the lack of improvement under TMR was probably not due to a ceiling effect. In contrast, for indirect associations (where we had observed an overall effect of TMR), the TMR trend remained for both the bottom and top half of participants (*p* = 0.071, top 50%; *p* = 0.034, bottom 50%). Together these findings suggest that the reported difference in effect of TMR on indirect and directly learned associations is likely not due to difference in task difficulty or due to a ceiling effect, but instead indicates that contextual TMR applied during awake rest prioritises formation of shortcuts that support inference, rather than strengthening learned memory content.

Together, these results suggest that while contextual TMR improves inferential choice across indirectly associated cues, this improvement cannot be explained by improved performance on directly learned associations. Therefore, the benefits of contextual TMR on inferential choice must be explained by a mechanism that goes beyond merely strengthening learned associations. This provides further evidence to suggest that periods of awake rest facilitate inference by restructuring knowledge and creating shortcuts between items in memory that extend beyond direct experience.

## Discussion

Previous studies have shown the benefit of rest/sleep for inference^9–13,36,37^. However, the nature of the changes that occur during rest/sleep to facilitate inference remain unclear. In this study, the central question we ask is: *How does memory reactivation reorganise knowledge during awake rest, to guide adaptive behaviour?* We sought to tease apart two ways in which rest/sleep could affect memories: an improvement in behavioural performance on an inference task could be supported by either a quantitative strengthening of learned, component associations (A → B and B → C), or a qualitative change, involving the formation of a new, ‘shortcut’ link (A → C). The latter, qualitative change would reflect an essential pre-requisite for the organization of knowledge into a structured cognitive map that is more than the sum of its parts.

Using a multi-stage inference task with awake auditory contextual TMR^29^, we show that memory reactivation during awake rest benefits inference by building a new shortcut (e.g. A → C), with no effect on directly trained associations (i.e. A → B). We demonstrate this result using two approaches. First, we show that TMR improves behavioural accuracy when participants are required to perform inferential choice (A → C). Second, using eye-tracking data we show that TMR reduces the time spent looking at the intermediary visual cues (B) during inferential choice. Together these results suggest that promoting memory reactivation during awake rest facilitates the formation of novel shortcuts in the cognitive map. This may explain why periods of rest and sleep facilitate behavioural readouts of inference, but also other cognitive processes such as insight, generalisation and abstraction^9–14^. Interestingly, both our behavioural and eye-tracking data show that these shortcuts cannot be as readily updated in response to subsequent changes in the environment. Rather, shortcuts (e.g. A → C) may limit behavioural flexibility when optimal behaviour requires participants to revert to a chain of learned associations (i.e. A → B, B → C). Together, our results reveal how memory reactivation during awake rest plays a causal role in *restructuring* memories, while highlighting how the organisation of knowledge sets an important trade-off between efficient and flexible behaviour. Thus, re-organising knowledge may improve behavioural flexibility, but only if the environment remains constant and the expected statistical relationships between events/cues are persevered.

While our ability to make novel inferences can clearly support efficient behaviour, representing inferred relationships as a shortcut (A → C) rather than a chain of learned associations (A → B, B → C) may limit behavioural flexibility. Our data demonstrates the limitation of building a shortcut in two key analyses. These two analyses are applied to data acquired after the value-flip manipulation, where half of the visual-outcome mappings are ‘flipped’ (B → C becomes B → C*). First, we show that the TMR benefit on performing inference is no longer observed for the Flipped cues. Second, for the Flipped cues, eye-tracking data shows that participants spend more trials looking at the previously correct, but now incorrect outcome (C*) for TMR compared to no-TMR cues. We note that after the value-flip manipulation overall performance remains high, suggesting that the full chain of learned associations (A → B, B → C*) are still recalled on the majority of trials. Together these findings suggest the following: first, the outdated shortcut (A → C) may compete with the updated chain of associations (A → B, B → C*); second, the shortcut (A → C) is indeed separate from the chain of associations (A → B → C/C*). Overall, these data demonstrate that shortcuts in memory (e.g. A → C) cannot be readily updated in response to rapid changes in the environment and may limit behavioural flexibility when optimal behaviour requires participants to revert to a chain of learned associations (i.e. A → B, B → C*). We speculate that if a further rest period was included after the change in value of outcome (C), a new updated shortcut (A → C*) would have been formed and used. Indeed, there is evidence to suggest that rest and sleep may play a role in integrating new memories into existing knowledge^38–41^. Our findings complement this work by suggesting that adaptive re-organisation of the cognitive map cannot always occur ‘on the fly’ and may instead rely on offline periods of rest or sleep. Our study therefore provides insight into how new information is incorporated into a cognitive map, to guide adaptive behaviour.

A candidate neural mechanism that may explain our results is replay. Previous studies suggest that auditory TMR leads to memory reactivation and replay in hippocampus^18,30^, but also in brain regions typically recruited during the initial learning phase^31,33,42^. Moreover, the power of particular oscillatory dynamics associated with memory consolidation are typically enhanced during TMR, along with coupling between encoding regions and hippocampus. In this study, memory reactivation in response to TMR may similarly drive memory reactivation in brain regions that represent cues in the inference task, together with hippocampal circuits. During an inferential learning task in mice, similar to the one employed here, hippocampal replay during rest/sleep was shown to go beyond direct experience by “joining-the-dots” between indirectly linked cues^26^. Specifically, during awake sharp-wave ripples (SWRs), hippocampal spiking activity increasingly represented the inferred relationship (A → C) in the absence of the intermediary cue (B). This contrasted with activity in the hippocampus at the time of inferential choice, where hippocampal activity appeared to draw directly from learned associations, representing the intermediary cue (B) but not the outcome (C)^26^. During inferential choice, the inferred outcome was found to instead be represented by prefrontal cortex (PFC) and putative ventral tegmental area (VTA)^26^. Taken together, this suggests a mechanism whereby spiking sequences in hippocampal SWRs may both strengthen directly learned component associations while also providing a training signal to other brain regions, to assign credit to cues that are indirectly associated with reward, thereby generating a short-cut.

However, in our previous work^26^, the behavioural consequences of this candidate mechanism were not tested. Here, in humans, we use a contextual TMR manipulation^29^ to bias the content of memory reactivation during awake rest. This causal manipulation suggests that memory reactivation during awake rest leads participants to form shortcuts between indirectly related cues. Furthermore, these shortcuts reduce the need to rely on directly learned associations, by reducing dependence on the intermediary cue. Together these findings suggest that our TMR manipulation promotes the formation of a shortcut in the underlying cognitive map by biasing the content of hippocampal replay such that inferred relationships in the TMR group are prioritised. Yet, we speculate that the shortcut may not be represented in hippocampus, despite offline hippocampal activity likely being necessary for construction of the shortcut. Since this study did not directly measure replay, future investigations will need to validate this interpretation using neuronal recordings.

When designing the multi-stage inference task, we leveraged a sensory preconditioning paradigm^2^. Sensory preconditioning is a well-established behavioural model of associative inference in both humans and animals^26,43–47^ and causal manipulations demonstrate hippocampal dependence^26^. Thus, while sensory preconditioning does not provide a model for episodic memory per se, the paradigm does aim to capture relevant features of episodic memory, such as associative relationships between stimuli, while showing necessary involvement of a common hippocampal circuit. Importantly, performance on a sensory preconditioning task captures the quintessential features of behaviours that draw from a cognitive map, by testing both recall of learned associations but also inference across sensory cues that have not been experienced together. Behavioural performance may therefore be thought to draw from a cognitive map that links preconditioned cues (A) to reward (C). The task design is parsimonious given that inference is performed across groups of 3 cues, arguably the minimum number of cues that allow for inference. To further test the effect of TMR on both inference and value updating with sufficient controls in place, we included 12 sets of 3 cues. Overall, our task design ensures participants show reliable and efficient learning, while minimising unwanted effects attributed to memory load or forgetting.

It was beyond the scope of this study to test the effect of memory reactivation on formation of larger cognitive maps or maps that include sensory cues deriving from alternative sensory modalities. However, we predict that our findings should generalise to inferences that occur across longer sequences of cues, including those formed from cues that derive from modalities that differ to those included here (i.e. auditory, visual, monetary). We note that electrophysiological data from V4 in non-human primates suggests that the temporal position within a 7-element visual sequence influences the extent of memory reactivation, where more robust memory reactivation emerges for cues at the start and end of the sequence^48^. One possibility is that these temporal-position effects can be explained by formation of short-cuts in the hippocampal cognitive map. To address this knowledge gap there is a need for future studies to record from both hippocampus and sensory cortices on tasks that include longer sequences of cues.

The specifics of our TMR manipulation must be considered when comparing our findings with that of others. Previous studies demonstrate that TMR during periods of post-encoding sleep elicits a robust effect on memory retention (for example^30,35,49–55^). More variable results are reported when using TMR during periods of awake rest^29,30,35,49,50,56–60^, which may in part be explained by variation in the level of task engagement during the rest period^50,58^. Specifically, application of TMR in the awake state appears to show no benefit for memory when cueing is coupled with externally-orientated, attention demanding tasks^35,50^, and appears to prioritize weakly learned information^50,58,61^. Our results are congruent with these previous findings, in suggesting that strongly learned direct associations do not reliably benefit from memory reactivation in awake rest, while weaker non-directly trained associations that extend beyond direct experience are facilitated by TMR^29^.

Moreover, we note that our TMR protocol differs from the majority of TMR manipulations by including a contextual rather than punctate auditory cues to trigger memory reactivation. Punctate auditory cues may have several drawbacks, including the need to precisely time the cue to specific phases of sleep oscillations to see the best effects^62^. Furthermore, since punctate cues are associated with specific stimuli, they provide a limited tool to investigate how links across multiple stimuli are consolidated. The contextual TMR protocol used here provides a unique opportunity to investigate the effect of memory reactivation on a group of cues that together form the primitive for a map of associations. As described previously^29^, the aim of this TMR protocol is to capitalise on the endogenous neural dynamics of reactivation within a cognitive map, without interrupting or altering the natural sequence of replay, as may occur with the introduction of punctate cues. Arguably, this approach is more comparable to TMR studies that have used contextual odour cues in sleep^30,63,64^, however we apply the contextual cue during awake rest. Our data replicate our previous findings: memory reactivation during awake rest improves performance on new behaviours, such as associative inference, with no effect on behaviours that draw directly from learned information^29^. In addition, we demonstrate *how* memory reactivation during periods of awake rest influences the organisation of memories within the cognitive map, where memory reactivation plays a causal role in forming novel links that go beyond direct experience.

Overall, our results demonstrate that knowledge is subject to qualitative restructuring during offline periods of rest/sleep. During awake rest, stimuli that are reactivated (due to contextual TMR) are organized into cognitive maps through the creation of novel shortcuts between indirectly linked cues. These shortcuts are distinct from the original chain of learned associations and the behavioural benefit of forming a shortcut cannot be explained by quantitatively strengthening the component learned associations (since there was no benefit of TMR on these learned associations even in participants whose performance was well below ceiling). Furthermore, when the component learned associations are rapidly modified in response to changes in the environment, shortcuts are not updated. Indeed, an outdated shortcut can conflict or compete with an updated chain of associations to control decision making, illustrating that the two sets of associations are separate. Together our findings provide new insight into how integrated cognitive maps are formed during periods of awake rest. Moreover, we demonstrate the effectiveness of using awake contextual TMR as a tool to reveal how the resting brain re-organises memory to shape adaptive behaviour.

## Methods

### Participants

A total of 32 healthy participants were recruited for this study. 7 participants were excluded from analysis as they failed to reach the performance criterion during the initial associative learning phase (criterion: > 85% performance accuracy for each contextual group of cues). The remaining 25 participants were included in the analysis (mean age of 24.72 ± 6.35 years (standard deviation), range 19–40, 4 males). All participants had normal or corrected-to-normal vision. All experiments were performed in accordance with the Declaration of Helsinki and all relevant guidelines and regulations, and were approved by the University of Oxford ethics committee (reference number: R43593/RE013). All participants gave informed consent prior to participating in the study.

### Experimental set-up

Cues were presented on a 24-inch screen with a spatial resolution of 1920 × 1080 pixels (width × height), a background luminance of 0.5 (grey), and a refresh rate of 100 Hz. The approximate distance of participants from the screen was 64cm, which meant that one degree of visual angle corresponded to 40 pixels on the screen. Stimulus presentation was controlled using Psychophysics Toolbox-3^65^ in MATLAB (version R2015b). Eye movements were recorded in the testing phase of the experiment (after the rest period) using an eye-tracking camera (EyeLink®, SR Research) tracking both eyes at a rate of 1kHz.

### Inference task—overview

Participants performed an inference task (Fig. 1A,B). In the first stage of the task (‘associative learning’), participants learnt to associate punctate auditory cues with visual cues. The punctate auditory cues were naturalistic sounds from the BBC sound library (https://sound-effects.bbcrewind.co.uk/) and the visual cues were unsymbolic shapes^66^. Each visual cue was paired with three auditory cues. In the second stage of the task (‘conditioning’), these visual cues were then associated with either a pound coin (rewarded outcome) or a wood coin (neutral outcome, of no value). The cues were split into two sets, grouped according to the contextual background music that played throughout learning. For one of the two sets of cues, participants heard jungle music in the background and the punctate auditory cues were sounds you would hear in a jungle (e.g., animal noises). For the other group, participants heard café music in the background and the punctate auditory cues were sounds you would hear in a café (e.g., a cash register). In total, 12 auditory cues and 4 visual cues were included in each group and the relationship between the conditions (i.e., which context was learnt first, and which context was subject to TMR) was fully counterbalanced across participants. Since the visual cues had no direct meaning, they were randomly assigned to each group across participants.

### Behavioural protocol

The associative learning stage was designed to allow participants to learn associations between auditory and visual cues. This stage of the task included passive exposure and testing trials (Fig. 1C). In the exposure phase, on each trial a punctate auditory cue was presented for 1.5 s, immediately followed by presentation of a visual cue for 1.5 s, followed by an inter-trial interval (ITI) of 2 s. Each mini-block of exposure consisted of 12 trials (one presentation of each auditory-visual pair). Each mini-block of exposure was followed by a testing mini-block. On each trial in the testing mini-block participants heard an auditory cue for 1.5 s before being presented with 3 possible visual cues. Participants were then required to select the visual cue that was paired with the auditory cue they just heard, responding within 5 s using the keys ‘j’, ‘k’, and ‘l’. Once a choice had been selected, a black rectangle would highlight this choice for 0.5 s, followed by an ITI of 2 s. At the end of each testing mini-block, participants were given feedback on their average performance. Each block in the associative learning phase was made up of one exposure mini-block followed by one testing mini-block and consisted only of cues from one of the two groups of cues, with the relevant contextual background music playing throughout the block. Blocks alternated between the two contexts with a total of 8 blocks per context. The relationship between the conditions (i.e., which context was learnt first, and which context was subject to TMR) was fully counterbalanced across participants.

The conditioning stage was designed to allow participants to learn associations between visual and outcome cues. The blocks followed the same structure as the associative learning stage, including both passive exposure and testing trials (Fig. 1D). In each trial of the exposure phase, a visual cue was first presented for 1.5 s, immediately followed by the associated outcome for 1.5 s, followed by an ITI of 2 s. Each exposure mini-block consisted of 4 trials (one presentation of each visual cue), which was followed by a testing mini-block. In each trial of the testing mini-block, a visual cue was presented together with the two outcome cues for 1 s. After this 1 s, the visual cue would disappear but the outcome cues would remain on the screen and the participants had a further 3 s to make their choice (total of 4 s response period). Participants had to select which outcome they thought was associated with the visual cue using the keys ‘j’ and ‘l’. Once a choice had been selected, a black rectangle would highlight their choice for 0.5 s, followed by an ITI of 2 s. Participants were given feedback on their average performance at the end of each mini-block of 4 trials. Each block in the conditioning phase was made up of one exposure mini-block followed by one testing mini-block and consisted only of cues from one of the two groups of cues, with the relevant contextual background music playing throughout the block. Blocks alternated between each context with a total of 2 blocks per context. The relationship between the conditions (i.e., which context was learnt first, and which context was subject to TMR) was fully counterbalanced across participants.

The TMR manipulation was performed after the conditioning stage. During TMR, participants were instructed to rest for up to 60 min, during which participants were asked to alternate between resting with their eyes closed for 10 min, while remaining awake, and doing a jigsaw puzzle for 20 min. In total, they spent 20 min with their eyes closed and 40 min doing a jigsaw puzzle. During this awake rest period, the contextual soundtrack associated with the TMR group was played throughout. The contextual soundtrack used for TMR was fully counterbalanced across participants. To encourage participants to attend to the soundtrack, the soundtrack was turned on and off for periods of between 9 and 16 s, alternating with periods of silence. The length of each on and off period was selected from a random distribution.

After the TMR manipulation, participants were required to perform an inference test. During the inference test, participants were asked to infer the relevant outcome in response to each auditory cue.

Before beginning the first inference test, participants practised performing two tasks together using only two auditory cues, not taken from the task, and a 1-back task using digits rather than shapes. The practice consisted of up to 4 blocks with 24 trials per block. The timings of these practice trials matched the timings of the main inference test described below. The participants were not required to perform under time pressure as reaction times were not speeded.

We designed three different versions of the inference test which included distractor tasks designed to increase the load on visual working memory or general cognitive resources (Figure S1A). For all versions, participants were presented with an auditory cue and had to choose which of the two outcomes they thought was associated with the auditory cue (Fig. 2B). The auditory cue was presented together with the two outcome cues for 1.5 s. After 1.5 s, the auditory cue would stop but the outcome cues would remain on the screen and the participants had a further 3 s to make their choice (total of 4.5 s response period). Participants had to select which outcome they thought was associated with the auditory cue using the keys ‘j’ and ‘l’. Once a choice had been selected, a black rectangle would highlight this choice for 0.5 s, followed by an ITI of 1 s. Participants were told they would receive a bonus reward of up to £5 based on how many cues they correctly inferred. Participants were given feedback on their average performance at the end of each block. Auditory cues from both contextual groups were randomly interleaved in each block and there was no background music during the inference tests.

For 2 of the 6 blocks, the participants also performed a visual memory distractor task (Figure S1A). The purpose of this distractor task was to modulate task difficulty: a visual memory distractor task could impair recall of the intermediate visual cue when participants perform inference. The visual memory distractor task involved using a 1-back task with shapes similar to those seen in the task. On each trial, between presentations of each auditory cue, a shape would appear for 2 s, and the participants had to respond based on whether it was the same or different from the previous shape using the keys ‘a’ and ‘d’. The shape would become darker (participant chose ‘same’) or lighter (participant chose ‘different’) after the participant had made their choice. The shape remained on screen until the 2 s was complete. The shapes would appear in a random orientation which participants were instructed to ignore. Following a pause of 1 s, the auditory cue would begin, and the rest of the inference trial would commence as described above.

For another 2 of the 6 blocks, participants performed a non-memory-based distractor task (Figure S1A). The purpose of this distractor task was again to modulate task difficulty, but without the interfering effect of a visual memory load. On each trial, between presentations of each auditory cue, an image would appear for 2 s, and the participants had to respond based on whether the image depicted something living (e.g., a hedgehog) or something non-living (e.g., balloons). The image would become darker (participant chose ‘living’) or lighter (participant chose ‘non-living’) after the participant had made their choice. The image remained on screen until the 2 s was complete. Following a pause of 1 s, the auditory cue would begin, and the rest of the inference trial would commence as described above.

For the remaining 2 blocks, there was no distractor task. After the normal ITI of 1 s, there would be an additional pause of 3 s to ensure trials across all versions of the inference task were the same length. Following this pause, the auditory cue would begin, and the rest of the inference trial commenced as described above. These 2 blocks which did not include distractor tasks always occurred at the end to minimise order effects of participants practising the inference task on its own.

After all 6 blocks of the first inference test, participants redid the conditioning stage of the experiment but with half of the associations between visual and outcome cues flipped (‘value flip’) (Fig. 3A). The protocol for the value-flip stage was otherwise the same as for the conditioning stage (Fig. 1D) except participants completed 1 block per context (rather than the 2 blocks per context done in the initial conditioning stage). After the value-flip, a second inference test (‘inference test 2’) was conducted using the same procedure as previously described and also included 6 blocks (Fig. 2B; Figure S1A).

In the indirect/direct test, we tested both the indirect (auditory–outcome) and direct (auditory-visual) associations paired with each auditory cue (Fig. 4B). All visual cues and outcomes were present on the screen. By having all cues on the screen, we could test whether there were differences in how long participants spent looking at the visual cues during inference in the TMR and no TMR groups. However, by displaying the visual cues during the indirect/direct test participants were provided with an opportunity to solve inference for all auditory cues (A) using the intermediary visual cues (B), rather than relying on a short-cut (A → C) generated during memory reactivation. Therefore, to test the effect of TMR on inferential choice it was necessary to only test inferential choice in the presence of the visual cues at the very end of the experiment, after the value-flip manipulation. For the first 2 blocks of this test participants completed the indirect test: on each trial an auditory cue was presented, and participants had to select one of the two outcomes using the mouse. For the final 2 blocks of this test participants completed the direct test: on each trial the auditory cue was presented, and participants had to select one of the 8 possible visual cues which was paired with the sound. This allowed us to assess performance on the directly learnt associations as well as the indirect auditory-outcome association. In each block a total of 24 trials were included, with equal number of trials in the TMR and no TMR condition. For all blocks, the auditory cue was presented for 1.5 s. Following this, participants had 30 s to select either the outcome cue (first 2 blocks) or the visual cue (last 2 blocks) associated with the auditory cue. Participants made their selection by moving the cursor over the chosen cue and pressing ‘spacebar’ to confirm. Once a choice had been selected, a black rectangle would highlight this choice for 0.5 s, followed by an ITI of 1 s. Participants were given feedback on their performance at the end of each block.

### Eye-tracking data preprocessing

For 2 participants, no usable eye-tracking data was collected. The remaining 23 participants were all included in eye-tracking analyses. The conversion of the EyeLink® 1000 Edf files was done with the Edf2Mat Matlab Toolbox designed and developed by Adrian Etter and Marc Biedermann at the University of Zurich. Gaze position and pupillometry data were smoothed using a 50 ms Gaussian kernel. Blinks were detected from the smoothed pupillometry data and removed from the gaze position data. For each participant, the eye with the fewest missing samples was selected and used for all further analyses. The gaze position was epoched around the auditory cue onset for trials in the second inference test and for indirect trials in the indirect/direct test. For the comparison of Flipped cues (or Not Flipped cues), we analysed the percentage of trials where participants looked at the incorrect outcome cue, using a binary label to establish whether the incorrect outcome had been viewed on any given trial. To investigate whether participants relied on knowledge of the intermediary visual cue, we analysed the time spent looking at the visual cues in response to auditory cues in the TMR and no TMR groups on correct trials of the indirect test. Due to the large variation in the average time spent looking at the visual cues, we z-scored the fixation lengths across all trials for each participant. We further analysed the proportion of trials where participants looked at the visual cues in response to auditory cues in the TMR and no TMR groups on correct trials of the indirect test.

### Analysis and statistical tests

Analysis was conducted in MATLAB (version R2022a) and in Python v.3.6 (https://www.python.org/downloads/release/python-363/), using the Python packages DABEST^67^, scipy^68^, numpy^69^, matplotlib^70^, seaborn^71^, pandas^72^. Throughout this study, we used a bootstrap-coupled estimation of effect sizes^73^. For each estimation plot showing a difference between TMR and No TMR groups: the left panel shows the distribution of raw data points for the entire dataset, superimposed on plots reporting group means ± SEM; and the right panel displays the difference between the TMR group and the No TMR group, computed from 100,000 bootstrapped re-samples. For each estimation plot: black dot, mean; black ticks, 90% or 95% confidence interval; and filled curve: bootstrapped sampling error distribution. All p-values were estimated using bootstrapping, across 100,000 bootstrapped resamples. We used 90% confidence intervals (one-sided) for tests of difference between TMR and No TMR groups, with the prediction that TMR improves accuracy, unless noted otherwise. For any other tests, we used 95% confidence intervals (two-sided). All tests are performed on n = 25 participants (degrees of freedom (d.f.) = 24), except for all tests on eye data which were performed on n = 23 participants (d.f. = 22).

## Electronic supplementary material

Below is the link to the electronic supplementary material.

Supplementary Material 1

## Data Availability

The data and code used in this study will be made available via the MRC BNDU Data Sharing Plaform (hrps://data.mrc.ox.ac.uk) upon publication: the contact for this platform is Ben Micklem, Research Support Manager, at ben.micklem@bndu.ox.ac.uk.
