# Supplementary material for: Memory reactivation during rest forms shortcuts in a cognitive map
Source: Sci Rep. 2025 Jul 9;15:24724. doi: 10.1038/s41598-025-06742-y (PMC12241591; doi:10.1038/s41598-025-06742-y)
Supplement: Supplementary file 1 — Supplementary Material 1 [file 41598_2025_6742_MOESM1_ESM.docx]

**Supplementary Materials**

**
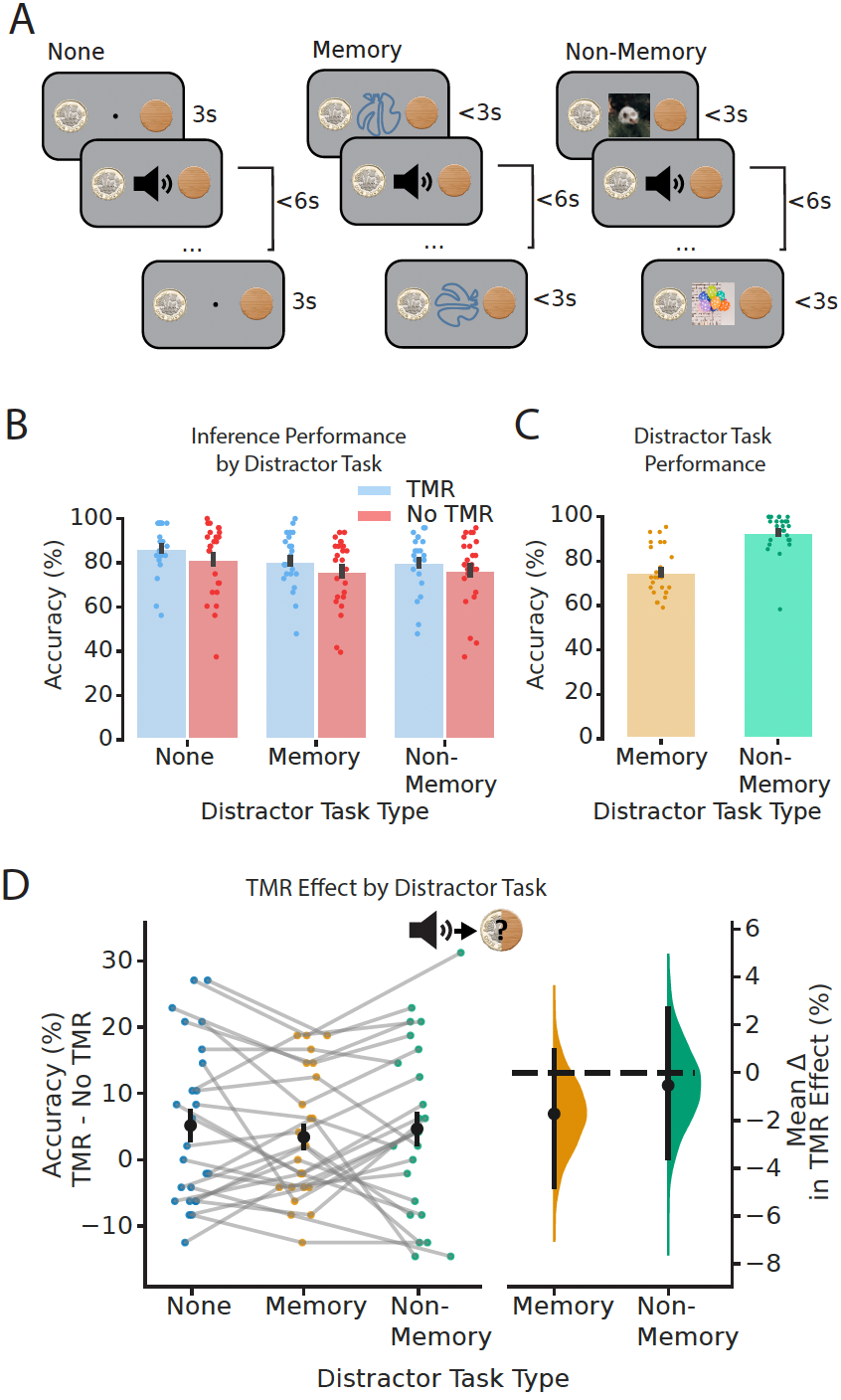
**

**Figure S1. Distractor Task Does Not Change the Effect of TMR On Inferential Choice.**
**(A)** Schematic: Example trials for different distractor task types during the inference test. Trials start with either no distractor task (left), a memory distractor task (participants are asked if a shape is the same or different to the one seen on the previous trial (middle)), or a non-memory secondary task (participants are asked to identify if a photo is of something living or non-living (right)). On all trial types, participants then hear an auditory cue and are asked to infer the outcome associated with that auditory cue. Full details of the inference part of each trial are shown in Figure 2B. **(B)** Inference test performance broken down by distractor task type and TMR group. Bars: mean percentage inference accuracy for no distractor task (left group), memory task (middle group), and non-memory task (right group) groups split by TMR (blue; left bar of each group) and No TMR (red; right bar of each group); black ticks: ± SEM; each data point: mean accuracy for one participant. The positive effect of TMR on accuracy is significant within each distractor task condition (p=0.013, no distractor task; p=0.031, non-memory task; p=0.031, memory task; all pairwise bootstrap tests for TMR vs no-TMR cues, one-tailed). **(C)** Distractor task performance. Bars: mean percentage accuracy on the distractor task for memory task (orange; left) and non-memory task (green; right); black ticks: ± SEM; each data point: mean accuracy for one participant. **(D)** Difference in TMR effect on inference between different distractor tasks. Right: raw data points for no distractor task (blue; right), memory task (orange; middle), non-memory task (green, right); each data point: mean effect of TMR (TMR group accuracy – No TMR group accuracy) for a given participant; black dots, mean; black ticks ± SEM. Right: difference in means between no distractor task (dashed black line) and other distractor task conditions shown using bootstrap-coupled estimation (DABEST) plots as in Figure 2C: black dots, mean; black ticks, 95% confidence interval; filled-curve, sampling-error distribution. There was no significant difference in the effect of TMR on accuracy between distractor task types (p=0.237, no distractor task vs memory task; p=0.741, no distractor task vs non-memory task – both pairwise bootstrap tests, two-tailed).


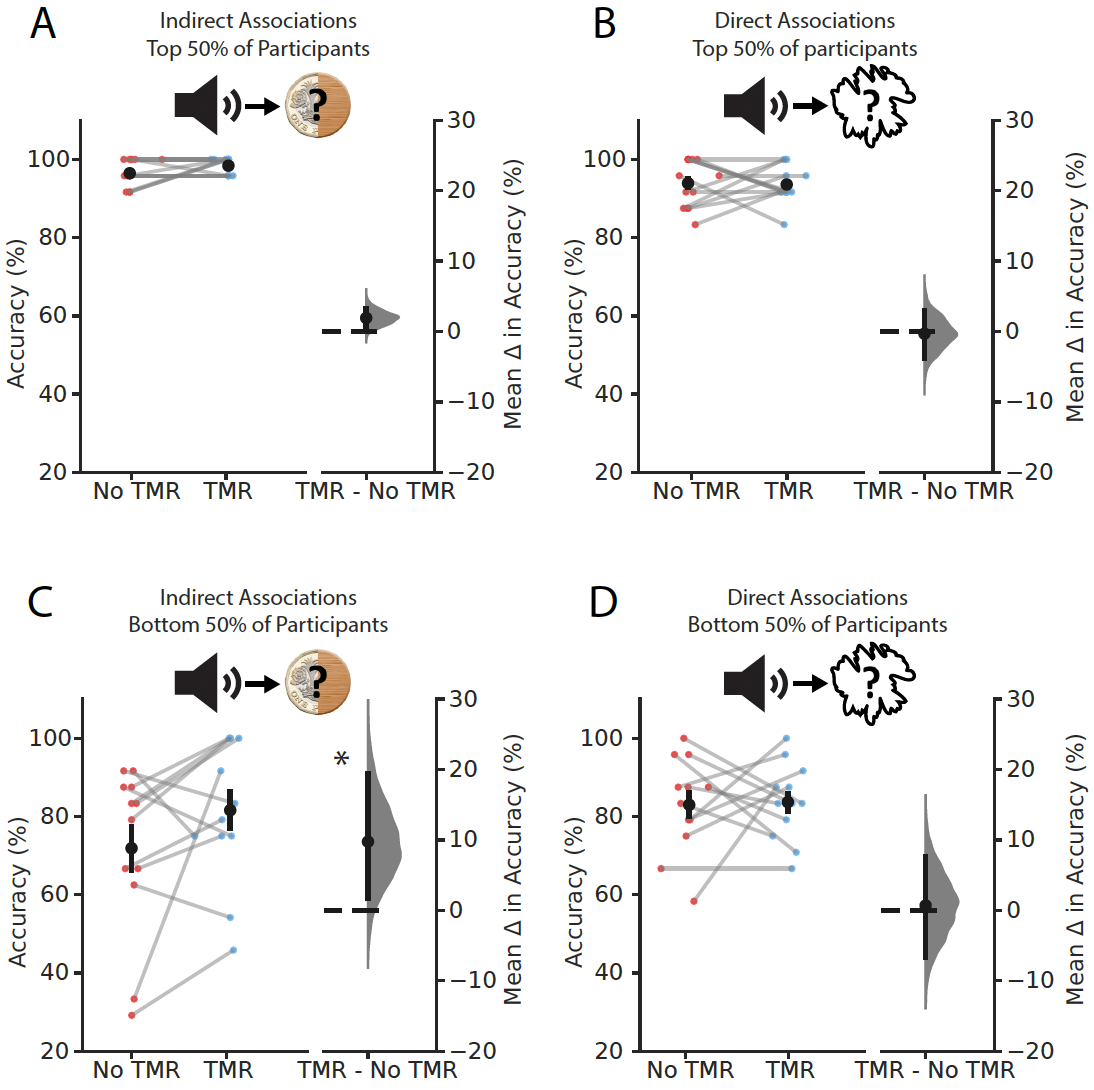


**Figure S2. Differences in Overall Accuracy Cannot Explain the Effect of TMR on Direct and Indirect Associations.**
**(A-D)** The effect of TMR on indirect associations (A; C) and direct associations (B; D) for participants split by overall accuracy in the top 50% (A-B) and bottom 50% (C-D). Left of each panel: raw data points for No TMR group (red; left) and TMR group (blue; right); each data point: mean accuracy for a given participant; black dots, mean; black ticks ± SEM. Right of each panel: difference in means between No TMR and TMR groups shown using bootstrap-coupled estimation (DABEST) plots as in Figure 2C: black dot, mean; black ticks, 90% confidence interval; filled-curve, sampling-error distribution. There was a positive effect of TMR on accuracy for indirect associations (i.e. inference) for the bottom (p=0.034, pairwise bootstrap test, one-tailed) and (n.s. trend) for the top (p=0.071, pairwise bootstrap test, one-tailed) 50% of participants. There was no effect of TMR on accuracy of direct associations, for either the bottom of top 50% of participants (p=0.389, top 50%; p=0.368, bottom 50%; pairwise bootstrap tests, one-tailed). Thus, the differential effect of TMR on indirect and direct associations cannot easily be explained by differences in performance accuracy or ceiling effects. Unsurprisingly, participants showed higher performance accuracy on direct compared to indirect associations. The median split analysis shows that both high and low performing participants continue to show a significant TMR effect for indirect but not direct associations. Therefore, participants who showed performance accuracy close to ceiling continued to show a significant TMR effect on indirect associations, while participants where performance was not at ceiling continued to show no significant effect of TMR on direct associations. Together these findings suggest that the differential effect of TMR on direct and indirect associations cannot be explained by differences in performance accuracy. * indicates p<0.05; n.s. indicates not significant.

***
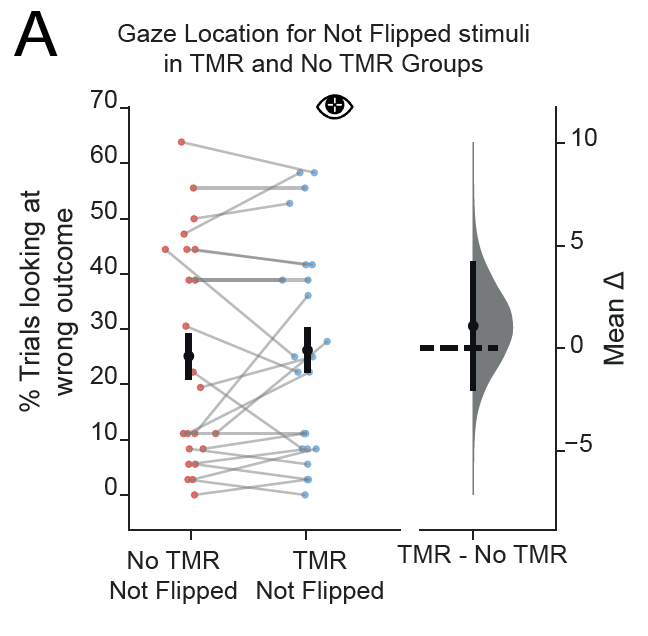
***

**Figure S3. Additional analyses of gaze data for Not Flipped cues.**

**(A)** For auditory cues where the visual cue-outcome association was not flipped (Not Flipped), gaze data revealed no significant difference in percentage of trials spent looking at the wrong outcome for auditory cues in the TMR compared to the No TMR group (p=0.276, paired bootstrap test, one-tailed). Analysis applied to correct trials only. Left: raw data points for No TMR group (red; left) and TMR group (blue; right); each data point: mean percentage of trials looking at wrong outcome for a given participant; black dots, mean; black ticks ± SEM. Right: difference in means between No TMR and TMR groups shown using bootstrap-coupled estimation (DABEST) plots as in Figure 2C: black dot, mean; black ticks, 90% confidence interval; filled-curve, sampling-error distribution.


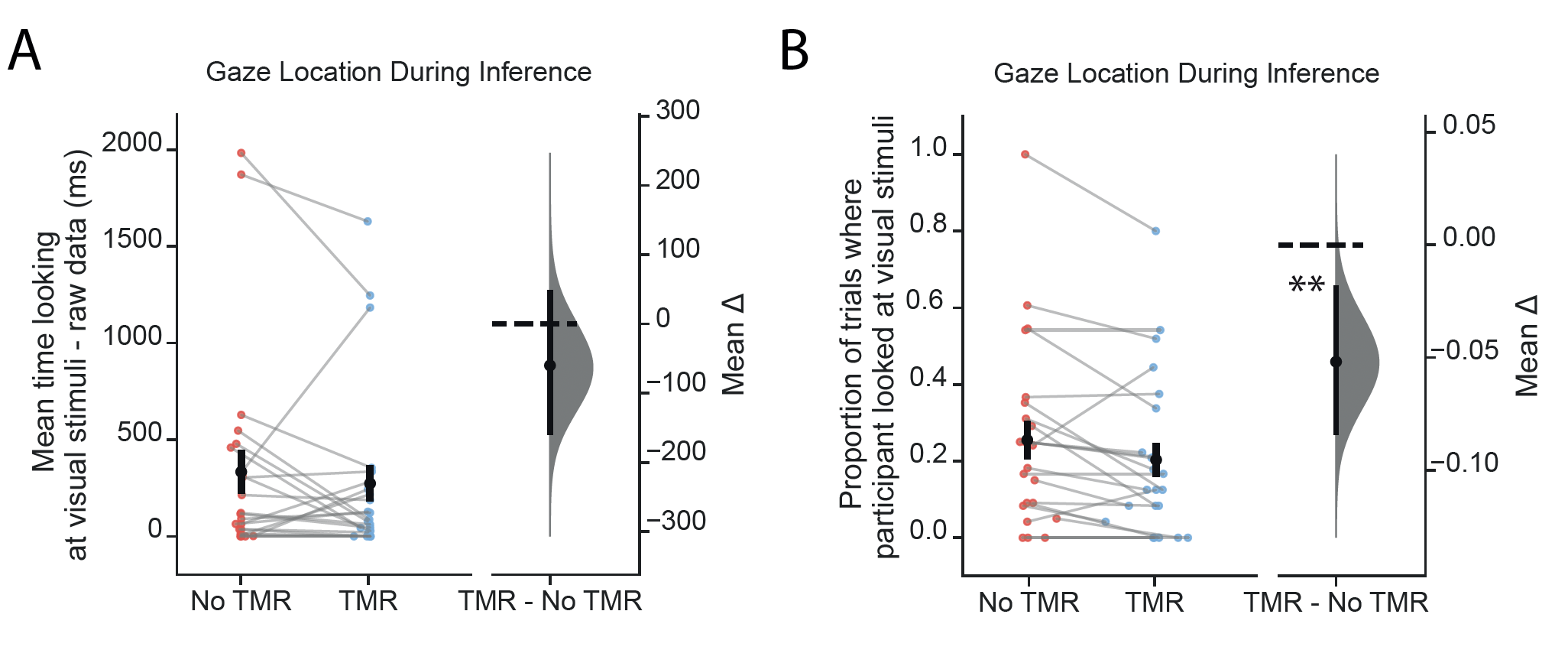


**Figure S4. Additional analyses of gaze data during inference, to support Figure 4C**

**(A)** Raw gaze location from eye tracking data acquired during indirect trials in the indirect/direct test, showing mean absolute time in ms spent looking at the visual cues during the indirect (inference) test. Due to high between-subject variance in time spent looking, a significant difference between cues in TMR and no TMR condition was not observed (p=0.179, pairwise bootstrap test, one-tailed; analysis applied to correct trials only). After normalising the data by z-scoring the time spent looking at the intermediary visual cues, a significant difference between TMR and no TMR was observed (p=0.014, pairwise bootstrap test, one-tailed, as shown in Figure 4C; population mean ± SEM: TMR condition, 334 [±110 ms,](https://wumbo.net/symbols/plus-minus/) no TMR condition, 274 [±91 ms](https://wumbo.net/symbols/plus-minus/)). **(B)** Raw gaze location from eye tracking data acquired during indirect trials in the indirect/direct test. Participants looked at the visual cues on a greater proportion of trials in the TMR compared to the no TMR conditions (p=0.006, pairwise bootstrap test, one-tailed; population mean percentage: TMR condition, 20.3%; no TMR condition, 25.5%). **(A-B)** Left: raw data points for No TMR group (red; left) and TMR group (blue; right); each data point: mean time spent looking at visual cues during inference (correct trials only); black dots, mean; black ticks ± SEM. Right: difference in means between No TMR and TMR groups shown using bootstrap-coupled estimation (DABEST) plots as in Figure 2C: black dots, mean; black ticks, 90% confidence interval; filled-curve, sampling-error distribution. ** indicates p<0.01
